# The therapeutic effects of chamomilla tincture mouthwash on oral aphthae: A Randomized Clinical Trial

**DOI:** 10.4317/jced.51472

**Published:** 2014-12-01

**Authors:** Seyyed-Amir Seyyedi, Majid Sanatkhani, Atessa Pakfetrat, Pooya Olyaee

**Affiliations:** 1 Assistant Professor. Faculty of Dentistry, Urmia University of Medical Sciences, Urmia, Iran; 2 Assistant Professor. Faculty of Dentistry, Mashhad University of Medical Sciences, Mashhad, Iran; 3 Associate Professor. Faculty of Dentistry, Mashhad University of Medical Sciences, Mashhad, Iran

## Abstract

Introduction: Recurrent aphthous stomatitis (RAS) is a common clinical condition producing painful ulcerations in the oral cavity. However, there has been no optimal therapeutic approach. Topical and systemic steroids commonly prescribed for the condition have local and systemic side-effects. Recently, there is growing tendency toward herbal medication in the modern society. The objective of this study was to assess the efficacy of a chamomilla mouth rinse on reducing the signs and symptoms of aphthous lesions in comparison with a placebo mouth rinse. Material and Methods: A randomized, triple-blind, placebo-controlled trial was performed on 36 patients, from cases diagnosed with RAS, attending the Department of Oral Medicine, Mashhad University of Medical Sciences. They were randomly divided into two groups: the intervention group(A), receiving chamomilla mouth rinse, and the control group (B) receiving a placebo rinse. The ability of the solution to control the pain and burning sensation and the number and size of the ulcers were evaluated. Results: The number of ulcers in the 3rd visit (four days after treatment) showed a significant difference between the groups (P<0.001). The pain and burning sensation (VAS) was reduced significantly in the test group in the 2nd (p=0.001),3rd and 4th visit (P<0.001). Conclusions: Chamomilla mouth rinse was effective in the treatment of RAS, controlling the pain and burning sensation without producing any adverse side effects and can be advised as an alternative RAS treatment.

** Key words:**Recurrent aphthous stomatitis, chamomilla mouth rinse, matricaria chamomilla, aphthous lesion treatment.

## Introduction

Recurrent aphthous stomatitis [RAS] remains the most common ulcerative disease of the oral mucosa, in the form of painful round, shallow ulcers with well-defined erythematous margin and yellowish-gray pseudomem-branous center (1). The etiology of RAS lesions is unknown, but several local, systemic, immunologic, genetic, allergic, nutritional, and microbial factors have been proposed as causative agents (2).

The proper treatment of RAS depends on the frequency, size, and number of the ulcers, and remains mostly palliative (3). The best treatment is the one to control ulcers for the longest period with minimal adverse side effects. The treatment approach should be determined by disease severity [pain], the patient’s medical history, the frequency of flare-ups, and the patient’s ability to tolerate the medication (3).

For common forms of RAS, standard topical treatment options that provide symptomatic relief include analgesics, anesthetics, antiseptics, anti-inflammatory agents, steroids, sucralfate, tetracycline suspension, and silver nitrate (4).

Complementary and alternative medicines are frequently used in chronic conditions (5). Chamomilla tincture is a herbal medicine which has many applications. The anti-inflammatory, anti spasm, anti bacterial, antifungal, and analgesic effects of chamomilla tincture have been investigated on inflammatory dermal lesions (6). Chamomilla mouth rinse is prescribed for aphthous lesions, gingivitis, and laryngitis (7). Faster healing has been observed after administration of chamomilla. The effective ingredients in Chamomilla are essences [azolene, camozolene] and flavonoids. Camazolene has a dose-dependent antiinflamatory and anti spasm effects, it inhibits leukoterine B4 [LTB4] synthesis, and peroxidation of arachidonic acid in neutrophils. Azolene has analgesic and anti-inflammatory effects. Flavenoids are also known to have anti-inflammatory characteristics (6).

There are few randomized clinical trials on the effect of chamomilla on aphthous lesions. The aim of this randomized clinical study was to investigate the therapeutic effects of chamomilla tincture mouthwash on oral aphthae.

## Material and Methods

In this triple-blind [patient, doctor, and statistics expert] randomized clinical study 36 patients, diagnosed with aphthous lesions were selected from the patients attending the department of oral medicine in Mashad faculty of dentistry between the years 2008-2010. All the patients were educated by the clinicians about their condition and the study, and then signed an informed consent approved by the ethical committee of Mashad Universityof medical sciences. A complete patient file was completed for each patient, in three parts; 1- demographic data [age, sex, educational degree,…], 2- complete medical history 3- questions about the aphthous lesions [ number of lesions, site, size, shape, degree of pain, past medication]. All the patients had their blood samples tested for: CBC, SI, TIBC, FE, Ferritin, and FBS.

Inclusion Criteria: confirmed clinical diagnosis of aphthous lesions, minimum of 2 weeks past from the last medications for aphthous. Exclusion criteria: Any systemic conditions or medication having interference with chamomilla/placebo mouth rinse, immunosuppressive medication, patients with anemia, blood dyscrasia, liver diseases, kidney diseases, GI tract disturbances, epilepsy, psychosis, all of which may develop aphthous-like lesions, pregnancy, lactation, age<10 years, warfarin uptake, syndrome-related aphthous lesions, e.g. Behcet, Crohn, ulcerative colitis,…, patients whose lesions did not regress after one month according to the standards defined in the literature (6) 50 patients with aphthous lesions were selected and randomly divided into group A [n=25], and group B [n=25], using the site “www.researchrandomiser.com”. Chamomilla tincture [Matrica Drop, Barij Esans, Iran] and its placebo, made by Mashad herbal medicine pharmacological research center, were used in this study. Chamomilla tincture and the placebo were coded as A, and B respectively. Half of the sample received drop A, and the other half received drop B, randomly. The drug and placebo had identical bottles, and the patient, clinician, and statistical expert were not aware of the medicine type [triple blind]. The patients were instructed to apply the drop three times a day [tid], and ten drops each time. Patients washed their mouth, and then rinsed for three minutes each time, and dischargedthe mouth rinsed refrained from eating for half an hour. The patients were clinically examined after 2, 4, and 6 days, and then on a weekly basis [if the lesions were still present]. Healing duration, number of lesions in each visit, size [lesser/greater than 1cm in diameter, measured by a digital caliper], side effects of the medication [the mucosa was investigated for any signs of side effected, and the patients were questioned regarding any burning or irritation symptoms], pain and burning were recorded using a visual analogue scale. If new aphthous lesions developed, they were separately recorded and followed up until complete healing. The patients were asked not to use any other medication in the study period without informing the clinicians.

Normality of data distribution was tested with Smirnov-Kolmogrov test. Independent t-test, and Man-Whitney tests were used [when the distribution was normal, and not-normal respectively] to compare the test and control group. For qualitative [ranking] data Man-Whitney and for qualitative [Nominal] data chi-square was used. *P* value < 0.05 was considered to be significant.

## Results

Total number of 36 cases fulfilled the study period, 4 cases in the case and 10 cases in the control dropped out of the study.

Comparison of mean age using Pearson Chi Square revealed that the sample was homogenous regarding age and there was no significant difference between the groups regarding age ( Table 1).

**Table 1:**
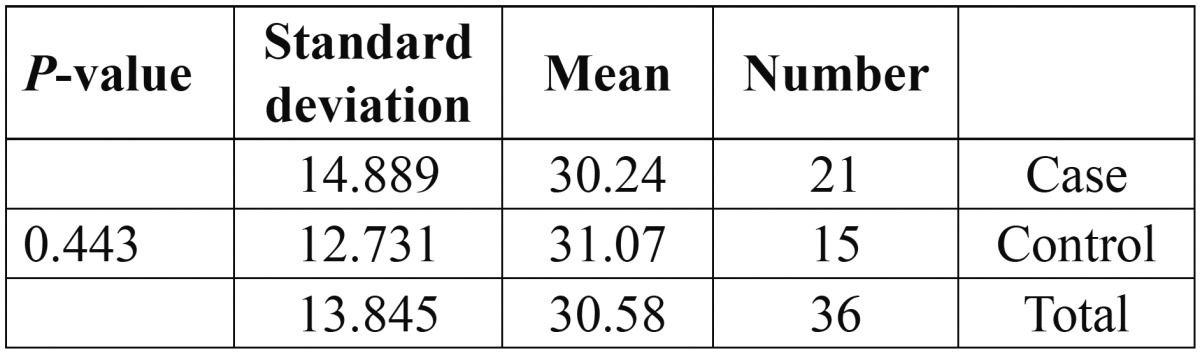
Comparison of mean age between the groups using Pearson Chi Square revealed that the sample were homogenous regarding age.

The mean number of lesions at the beginning of the study was 2.95 in the case group and 2.67 in the control group, Pearson chi square test revealed no significant difference and the groups were homogenous ( Table 2).

**Table 2:**
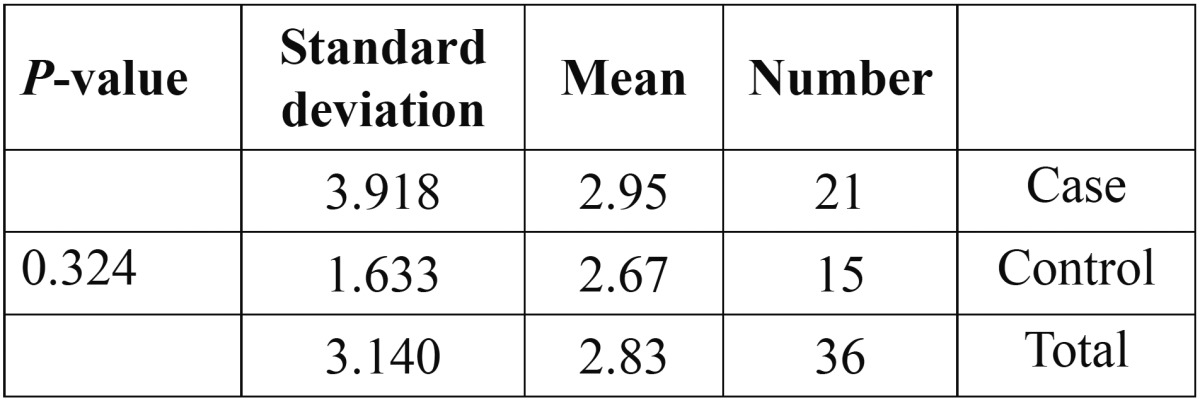
Comparison of mean number of lesions between the groups using Pearson Chi Square revealed that the sample were homogenous regarding number of lesions.

Moreover, Pearson chi square test revealed no significant difference between the groups regarding sex, pain and burning sensation, and the site of the lesions.

Mean number of the lesions in the second visit was 1.62 in the case group and 2.47 in the control group. Man Whitney test revealed a statistically significant difference [*p*=0.025]. Regarding pain and burning sensation in the second visit [using visual analogue scale], it was 2.9 in the case group and 6.3 in the control group. T-test revealed a statistically significant difference [*p*=0.001]. Lesion size was also significantly smaller in the second visit in the case group as shown with K2 Pearson test [*p*=0.03]

Mean number of the lesions in the third visit [4 days after initiation of the study] was 0.61 in the case group and 2 in the control group. Man Whitney test revealed a statistically significant difference [*p*<0.001]. Regarding pain and burning sensation in the 3rd visit [using visual analogue scale], it was 0.5 in the case group and 4.8 in the control group, the difference was statistically significant [*p*<0.001]. Lesion size was also significantly smaller in the 3rd visit in the case group as shown with K2 Pearson test [*p*=0.018]

Mean number of the lesions in the fourth visit [6 days after initiation of the study] was 0 in the case group which means all the lesions had healed and 1.3 in the control group. Man Whitney test revealed a statistically significant difference [*p*<0.001]. Regarding pain and burning sensation in the fourth visit [using visual analogue scale], it was 0 [no pain] in the case group and 3.4 in the control group, the difference was statistically significant [*p*<0.001]. Lesion size was also significantly smaller in the fourth visit in the case group as shown with K2 Pearson test [*p*<0.001].

**Figure 1:**
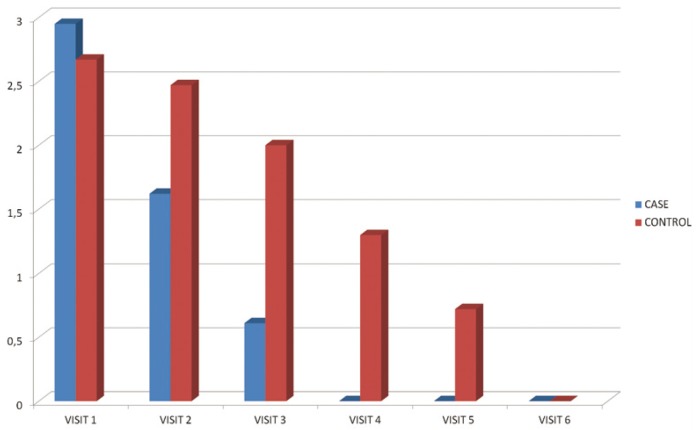
Mean number of lesions in different visits.

**Figure 2:**
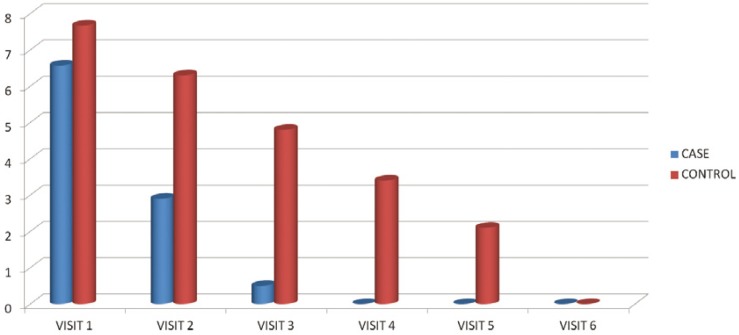
Mean pain and burning sensation in different visits.

Changes in the number of lesions and pain and burning sensations in the visits have been illustrated in figures 1,2.

## Discussion

The purpose of present study was to assess the efficacy of a chamomilla mouth rinse on reducing the pain, size and number of RAS in comparison with a placebo mouth rinse. The study had some advantages compared to the previous studies including being triple blind, matched groups [regarding age, sex, number of lesions, pain and burning sensation as reported by the patients, lesion site, size and form] which makes comparisons more reliable.

The investigated items each visit [number of lesions, pain and burning sensation, and lesion size] showed significant regression in the 2nd, 3rd, and 4th visits, which indicates the efficacy of chamomilla in all the visits.

Signs and symptoms decreases after two days of chamomilla consumption in most of the patients in the test group. On the other hand in the control group 68% of the lesions were still present and symptoms had not relieved completely, while in the case group all the samples had complete healing. Regarding the self-limiting nature of aphthous lesions, the important point in administration of different drugs is the speed of healing, which was prominent in the chamomilla mouth rinse group. Chamomilla significantly reduced healing period, number of lesions, pain and burning sensation.

Two days after chamomilla rinsing pain and burning sensation relieved significantly [*p*=0.001], compared with the control group. The size and diameter of the lesions decreased significantly in the 2nd, and 3rd visits, in the study group.

Ramos-silva *et al.* have evaluated the fluidextract from Chamomilla recutita’s effectiveness in pain relief from aphthous stomatitis and other painful ulcers of the oral mucous membrane, 15 minutes after consumption of the medicine. They found analgesic effects, and proposed chamomilla as a medication to improve the quality of life of RAS patients. The analgesic quality was also reported by the patients in this study (6).

Duart *et al.* evaluated the effect of Chamomilla recutita on the healing of ulcers in rats. Chamomilla stimulated re-epithelialization and the formation of collagen fibers after 10 days of treatment (8). Their result might cautiously explain accelerated healing of aphthous lesions in our study in the case group, in 100% of the cases after 4 visits. Although animal studies have shown accelerated healing after chamomilla administration (8,9), their results cannot be simply generalized to the human population.

Regarding the self-limiting nature of RAS lesions, even in the placebo group, the lesions finally regressed, but the point is that in the chamomilla group the healing process was accelerated, and from the second visit the symptoms were vanished, but the placebo group showed the normal healing process of a lesion.

Chamomilla is an effective agent in RAS condition, and can be administered whenever appropriate. Future research should aim at clinical trials comparing chamomilla with corticosteroids.
